# 

**Published:** 1902-10-04

**Authors:** 


					f-Jtyy- "Wisp
the hospital. (/>"-" "The standard of highest purity??lancet, Oct. 4th, 1m
CADBURY'S ii w CADBURY'S:
COCOA Fll m OOOOA r
ABSOLUTELY PURE. JB&ji NO DRUGS OR ALKALIES UBBD.
Lasqoit Weetern Infirmary
go. 836. Vol, XXXIII. [a Newspaper]
Saturday, Oct. 4th, 1902.
[SubBoriptionTenn*,] pRICH TWOPENCE.
AUTUMN SPECIAL NUMBER.

				

